# Assessing the global variation in patient characteristics, management and short-term outcomes of spontaneous intracranial haemorrhage worldwide: a protocol for a global observational prospective multicentre study (the PLOT-ICH study)

**DOI:** 10.1136/bmjopen-2025-100361

**Published:** 2025-09-02

**Authors:** Sara Venturini, David Clark, Brandon George Smith, Laura Hobbs, Michael F Bath, Harry Mee, Megan Still, Saniya Mediratta, Mohamed Amin Soliman, Katharina Kohler, Charlotte Jane Whiffin, Elijah Katambo, Tommi K Korhonen, Sami Tetri, Nourou Dine Adeniran Bankole, Nicephorus Rutabasibwa, Arnold Bhebhe, Thangaraj Munusamy, Abenezer Tirsit, Kee B Park, Karol Budohoski, Petra Wahjoepramono, Arina Tamborska, Deanna Saylor, Tariq Khan, Almas Fasih Khattak, Anthony Figaji, Angelos G Kolias, Tom Bashford, Alexis Joannides, Michael Martin, Rikin A Trivedi, Peter J Hutchinson, on behalf of the PLOT-ICH Collaborative

**Affiliations:** 1 NIHR Global Health Research Group on Acquired Brain and Spine Injury, University of Cambridge, Cambridge, UK; 2 Department of Neurosurgery, University of Florida, Gainesville, Florida, USA; 3 Cairo University, Cairo, Egypt; 4 University at Buffalo, Buffalo, New York, USA; 5 College of Health of Social Care, University of Derby, Derby, UK; 6 Neurosurgery Department, University of Zambia University Teaching Hospital, Lusaka, Zambia; 7 Department of Neurosurgery, Neurocenter, Oulu University Hospital, Oulu, Finland; 8 Centre Hospitalier Régional Universitaire de Tours, Tours, France; 9 Department of Neurosurgery, Muhimbili Orthopaedic Institute, Dar es Salaam, Tanzania; 10 University of Malaya, Kuala Lumpur, Malaysia; 11 Tikur Anbessa Hospital, Addis Ababa, Ethiopia; 12 Program in Global Surgery and Social Change, Harvard Medical School Department of Global Health and Social Medicine, Boston, Massachusetts, USA; 13 Department of Neurosurgery, University of Utah Health, Salt Lake City, Utah, USA; 14 Universitas Pelita Harapan, Tangerang, Indonesia; 15 Department of Epidemiology, Harvard T H Chan School of Public Health, Boston, Massachusetts, USA; 16 Northwest General Hospital and Research Center, Peshawar, Pakistan; 17 University of Cape Town, Rondebosch, Western Cape, South Africa; 18 Obex Technologies, Cambridge, UK

**Keywords:** Stroke, Brain Injuries, Intracerebral Hemorrhage

## Abstract

**Introduction:**

Stroke is the second leading cause of death worldwide, with the greatest burden in low- and middle-income countries (LMICs). Haemorrhagic stroke or spontaneous intracranial haemorrhage (sICH), including intraparenchymal haemorrhage (IPH) and subarachnoid haemorrhage (SAH), has the highest mortality and morbidity. Local management practices for haemorrhagic stroke vary greatly between geographical regions. The Planetary Outcomes after Intracranial Haemorrhage study aims to provide a global snapshot of the patient characteristics, processes of care and short-term outcomes of patients being treated for sICH across high- and low-income settings. It will also describe variation seen in care processes and available resources and time delays to receiving care. A greater understanding of the current state of sICH care is essential to identify possible interventions and targets for improved standards of care in all settings.

**Methods and analysis:**

We describe a planned prospective, multicentre, international observational cohort study of patients admitted to hospital for management of sICH. We will include patients of all ages presenting to hospital with imaging evidence of sICH (IPH, intraventricular haemorrhage and/or SAH). The study will collect patient, care process and short-term outcome data, following patients for up to 30 days (or until discharge or death, whichever occurs first). Any centre globally where patients with sICH are admitted and managed can participate, targeting a sample size of 712 patients. The study will recruit centres worldwide through pre-existing research networks and by dissemination through neurosurgical and stroke conferences and courses. Each participating centre will complete a site questionnaire alongside patient data collection.

**Ethics and dissemination:**

The study has received ethical approval by the University of Cambridge (PRE.2024.070). Participating centres will also confirm that they have undergone all necessary local governance procedures prior to starting local data collection. The findings will be disseminated via open access peer-reviewed journals, relevant conferences and other professional networks and lay channels, including the study website (https://plotich.org/) and social media channels (@plotichstudy).

**Trials registration number:**

NCT06731751.

**Strengths and limitations of this study**

- The Planetary Outcomes after Intracranial Haemorrhage study will use a collaborative research methodology to provide a global snapshot of current standards in care of patients with spontaneous intracranial haemorrhage (sICH), from the time of admission to the time of discharge or 30 days.
- The data fields will include both patient and disease characteristics and care process information, providing information on clinical outcomes and healthcare system processes.
- The study protocol has been developed by an international multidisciplinary panel of experts in brain injury (including stroke and neurovascular surgery), with extensive input from patient and public groups.
- Key limitations of the study are that only short-term outcomes are collected, and that only patients reaching hospital for treatment of sICH are included, due to the pragmatic study design.

## Introduction

 Stroke is the second leading cause of mortality worldwide and the third leading cause of death and disability combined.^1^ The global stroke incidence is reported at 12.2 million annually and is increasing, with over 80% of cases and associated morbidity and mortality occurring in low- and middle-income countries (LMICs).^1 2^ Stroke is a hallmark non-communicable disease that contributes significantly to the global disease burden and is now disproportionately affecting LMICs undergoing epidemiological transitions. There are now increasing concerns that this burden will continue to grow globally without reciprocated effective interventions.

Haemorrhagic strokes, that is, spontaneous intracranial haemorrhage (sICH), account for approximately one-third of the global stroke burden, with intraparenchymal haemorrhage (IPH) accounting for 27.9% and subarachnoid haemorrhage (SAH) accounting for 9.7% of all strokes, totalling a global estimated incidence of 4.59 million in 2019.^3^ Despite being the minority of cases, IPH and SAH are responsible for the highest morbidity and mortality, and disproportionately affect younger individuals.^3 4^ Geographical variations exist, with IPH being two times as common in LMICs as in high-income countries (HICs) (29.5% vs 15.8%), while SAH is more common in HICs (7.9% vs 19.7%).^1^ However, the true SAH incidence in LMICs is likely to be higher due to underreporting either because a significant proportion of individuals are not reaching hospitals or due to lack of access to neuroimaging to confirm the diagnosis.^5^ Several modifiable risk factors contribute to haemorrhagic stroke, such as hypertension, high body mass index and smoking, as well as environmental factors, such as air particle pollution, meaning that multiple areas for intervention are available.^1^ Better understanding how these factors contribute to disease burden in different settings is important to develop targeted prevention strategies.

Management for sICH is multidisciplinary, with recent evidence suggesting that, in well-resourced healthcare settings, acute care bundles, including rapid access to imaging, blood pressure control, anticoagulant reversal and surgical referral for decision-making, improve outcomes.^6^ Processes of care, including diagnosis, referral and definitive treatment, vary depending on local pathways and resources. Diagnosis of underlying structural causes for the haemorrhage is essential, as this is more likely to require surgical intervention. This requires access to advanced neuroimaging, such as CT/magnetic resonance angiograms or formal cerebral angiograms to identify vascular abnormalities, such as aneurysms, arteriovenous malformations or dural arteriovenous fistulae and cavernomas. However, advanced diagnostics are not always available globally, resulting in diagnostic challenges, which, in turn, lead to missed opportunities for prompt treatment and prevention of disease sequelae.

Surgery (open surgical clipping or endovascular coiling) is often indicated in aneurysmal SAH.^7^ Endovascular treatments, which are becoming increasingly popular around the world, are not yet available in many resource-limited settings.^8^ Even when they are available in resource-limited settings, these interventions are particularly costly, further limiting their availability to only the wealthiest patients in these settings. Surgical clipping, if available, may be the only option in many LMICs.^9 10^ Even when available, delays to surgery can occur due to challenges in access to specialist services and resources, resulting in poorer outcomes than when surgery is performed without delay. Settings where neither treatment option is available are not uncommon in LMICs, and patients are, therefore, managed conservatively.^9^

The role of surgery in IPH remains debated, with significant variation even across high-resource settings.^11^ For example, in the UK, management is primarily conservative under the care of stroke physicians in specialised hyperacute stroke units, whereas in Japan, surgery (often using minimally invasive procedures) is increasingly used.^12 13^ The STICH and STICH II trials showed no benefit of early surgical evacuation but were criticised for strict inclusion criteria.^14^ A recent meta-analysis suggested possible benefits of (minimally invasive) clot evacuation early after symptom onset.^15^ The ENRICH trial showed that minimally invasive evacuation of a supratentorial lobar haemorrhage, when performed within 24 hours, is associated with better functional outcomes than medical management alone.^16^ For infratentorial IPH, such as intracerebellar haemorrhage, surgical evacuation is more common due to the posterior fossa compartment being more susceptible to mass effect.^17 18^

Despite a significant global sICH burden, there is still limited description of its full impact and outcomes, especially in LMICs. Understanding the worldwide variation in sICH-related morbidity, and how this relates to patient and health system factors, is needed to plan services and prioritise context-appropriate interventions.

### Aim

The Planetary Outcomes after Intracranial Haemorrhage (PLOT-ICH) study aims to provide a global snapshot of patient characteristics, processes of care and clinical outcomes of patients being treated for sICH in high-, middle- and low-income settings. It will also describe the variation in available treatment modalities and resources and time delays to receiving care between clinical settings.

### Objectives

Specific objectives are as follows:

- estimate sICH case volume to gain insight into variations in epidemiology across world regions;
- describe differences in patient baseline characteristics, presentation and risk factors;
- describe the variation in available resources and management pathways for sICH;
- describe the variation in non-operative and operative management strategies across income settings;
- describe preoperative, perioperative and postoperative processes of care (including temporal delays to care);
- identify possible indicators of high-quality care for sICH across income settings;
- identify gaps in the implementation of best practices along the existing local pathways and care standards;
- compare short-term clinical outcomes across high-, middle- and low-resource settings.

## Methods and analysis

### Study design

The PLOT-ICH study is a prospective multicentre observational cohort study of patients admitted to hospital for the management of sICH. A multicentre snapshot audit study design was chosen as this offers the opportunity to generate a large, diverse dataset representing many geographical regions, including those often under-represented in research, such as low-resource settings, as demonstrated by examples, such as the Global Neurotrauma Outcomes Study (GNOS), GNOS-Spine and GOAL Trauma studies.^19 20^ This approach encourages collaboration between centres worldwide, and a pragmatic data collection window (30 days) with a short follow-up period per participating centre facilitates contribution while minimising losses to follow-up and attrition related to prolonged data collection periods. These elements contribute to generating real-life data with findings that are impactful globally and delivered in a timely manner.

The core study team, comprising the steering committee and an advisory panel, who were involved in the study design and protocol writing, will oversee the study.

### Study setting and population

The study will recruit centres worldwide through pre-existing research networks and collaboratives, developed through the National Institute for Health and Care Research Global Health Research Groups on Neurotrauma and Acquired Brain and Spine Injury. Invitation to participate will also be extended through relevant professional societies and academic groups.

Any hospital admitting patients with sICH worldwide is eligible to participate. A minimum of one case must be submitted by each site during the 30-day chosen study period. At each site, the local team will comprise a local lead, up to two additional data collectors, a data validator and supervising clinician. The study recruitment is planned to run for a total of 12 months (February 2025–January 2026). Eligible patients presenting with sICH over a consecutive 30-day period chosen by each individual recruiting centre during the overall study period will be enrolled. Patients will be followed up until 30 days, death or discharge from hospital, whichever comes first. All data will be submitted to the central study team for inclusion in the final dataset. A minimum sample size of 712 patients will be recruited, as detailed in the Statistical Analysis section below.

### Patient and public involvement (PPI)

During the study protocol development, patient and public groups were involved. A PPI discussion took place, involving people with lived experiences of brain injury, relevant to the project. Individuals were consulted at multiple stages; they reviewed and provided feedback on written study documents, and a further online group discussion took place around the key study aims, data fields and outcomes. Feedback derived from this session was incorporated into and influenced aspects of the proposed methodology and outcomes. The PPI groups influenced the study design with regards to the overall study aims and study outcomes.

### Eligibility criteria

The study will include patients of any age meeting the case definition criteria and admitted to a participating institution with an sICH during the selected 30-day study period. The case definition is:

- patients of any age with the presence of sICH (IPH, intraventricular haemorrhage and/or SAH haemorrhage) on intracranial imaging, in the absence of a definite history of head trauma, or other clinical features highly suggestive of head trauma;
- hospital admission for sICH management. This includes admission for observation, conservative or interventional management to both ward-based and critical care settings.

A patient will be included in the final analysis if>70% of the data fields required have been recorded.

Exclusion criteria are as follows:

- patients who have a clear history of head trauma as the primary cause of the haemorrhage and, therefore, are diagnosed with traumatic intracranial haemorrhage;
- patients with subdural or extradural haematomas but no clear history of trauma;
- patients with intracranial haemorrhage occurring as a complication of a procedure or intervention (eg, postoperative haematoma) or iatrogenic (eg, after thrombolysis for ischaemic stroke);
- patients who are admitted for elective (planned admission to hospital) or semielective (where a patient was initially admitted to hospital, then discharged from hospital and readmitted for surgery) procedures;
- patients readmitted to hospital within 30 days of the initial admission with sICH (including those readmitted for acute management of sICH-related complications);
- patient presenting to the emergency department (ED) but discharged home from the ED immediately without a period of observation or admission to a ward.

### Data collection and storage

Data will be collected from consecutive patients who met the inclusion criteria during the study period. The dataset contains five sections: baseline data, imaging data, acute management data, operative or interventional data (if applicable) and outcome data (see online supplemental material). The data points were determined through consultation with clinicians from a range of income settings. Only pseudonymised data will be collected on the online platform, and anonymised data will be accessed by the central study team.

#### Outcome measures

The primary outcome of the study is 30-day mortality or survival to hospital discharge (whichever comes first). Participating centres will be stratified according to income settings, and mortality rates will be compared across cohorts defined by income groups.

Secondary outcome measures include:

- perioperative complications, including but not limited to surgical site infections;
- clinical outcomes other than mortality, measured through:
  - Glasgow coma scale at discharge;
  - modified Rankin scale.
- Length of hospital stay and intensive care unit stay.

Each unit will be asked to complete a questionnaire on care processes and resources available. This will describe local case load, catchment population and referral pathways, and management frameworks and infrastructure at prehospital and hospital level.

Data will be collected and stored exclusively on a secure web-based system within the Outcome Registry Intervention and Operation Network platform https://orion.net/. This will enable secure data collection, validation and storage in a standard format, which is compliant with the UK National Health Service (NHS) security standards (including the Information Governance Toolkit). All patient data will be recorded and held anonymously. A pilot phase was conducted to assess feasibility prior to rolling out the full study.

### Statistical analysis

A formal a priori statistical plan will be produced for data analysis. In short, the primary analysis will comprise statistical descriptive analysis of patient-level variables and the characterisation of processes of care using descriptions of provider profiling data and patient-level process variables.

Country groups will be defined using the human development index (HDI), a recognised index of human development.^21^ The 30-day mortality will be reported for each HDI tier. Further analyses will stratify against other indices of economic development and health. Multivariable logistic regression models will be used to investigate the primary and secondary outcomes. Provider profile summaries will be compared with current evidence-based best practices to identify gaps and associations with care processes and case mix.

There is limited robust epidemiological data on sICH globally across income settings, with mortality rates for hospital cohorts reported between 20% and 33% in HICs and 39% and 62% in LMICs.^22^,^28^ Therefore, an accurate sample size calculation is not feasible. However, taking conservative mortality estimates indicating a 30-day mortality rate for sICH of around 30% in HICs and up to 40% in LMICs, using an alpha value of 0.05 and power of 80% to show a 10% difference between HIC and LMIC settings, a total of 712 patients would need to be recruited. Subgroup analyses will be performed, for example, to describe differences between sICH subtypes or hospital type.

Based on the data and experience from other observational studies with a similar methodology, we would expect that each centre enrols around three to five patients over a 30-day study period.^19^ Therefore, with around 150 recruiting centres from our pre-existing research network, we would expect to meet and exceed the sample size required. Given the nature of this international study, missing values are expected and could affect some participating centres more than others. Patients with at least 70% data field completeness will be included in the analysis. At each site, a data validator, independent from the primary data collectors, will validate accuracy for key study variables to ensure completeness and data accuracy.

### Limitations

There are limitations worth noting in this study. First, long-term follow-up data are not feasible for this study design. Additionally, this pragmatic study will only include patients reaching hospital for their sICH and, therefore, cannot provide comprehensive data on population-level sICH disease burden. Data validation will ensure robustness of the dataset; however, some missing data are expected in this collaborative study. Finally, this study will not test the efficacy of specific management strategies or novel interventions for patients with sICH.

### Ethics and dissemination

The study received ethical approval from the University of Cambridge Psychology Research Ethics Committee (PRE.2024.070). Using the UK NHS Health Research Authority tool (https://www.hra-decisiontools.org.uk/research/),^29^ this study is considered locally as a clinical evaluation study in the UK and will need to be registered as such at each participating site. In this setting, no formal informed consent is required, as data fields relate to those routinely collected as a part of standard clinical care. All participating centres will confirm that they have undergone all necessary local governance procedures prior to starting local data collection. Ethical approval processes and regulations vary between countries and obtaining informed consent from participants may be needed in some settings. We have prepared patient information sheets and consent forms for participating sites requiring these, in accordance with local regulatory processes. We expect that many participating sites will have access to waived consent procedures for the type of data collected in this study, which has been the case for large observational studies with similar methodologies.^19 30^ All data will be anonymised before it is accessed by the central study team. The study has been registered to ClinicalTrials.gov (NCT06731751) and will be reported using the Strengthening the Reporting of Observational Studies in Epidemiology statement guidelines and checklists.^31^ The study website www.plotich.org can also be accessed for all study documentations.

A full report of the study will be produced and published in an open access peer-reviewed journal with global readership. We expect many collaborators in this study; a collaborative authorship model will be used for all publications derived from the study itself, as outlined in the Guidelines for Standardising Reporting of Authorship in Collaborative Research.^32^

Results will also be communicated through presentation at relevant conferences and meetings, including existing international collaborator network and societies. Each participating site will also have access to their local data for presentation at institutional level. Lay-facing summaries of the results will be produced and shared with relevant stakeholders, communities and patient groups.

## Supplementary material

10.1136/bmjopen-2025-100361 online supplemental file 1
