# Supplementary material for: Assessing the global variation in patient characteristics, management and short-term outcomes of spontaneous intracranial haemorrhage worldwide: a protocol for a global observational prospective multicentre study (the PLOT-ICH study)
Source: BMJ Open. 2025 Sep 2;15(9):e100361. doi: 10.1136/bmjopen-2025-100361 (PMC12406811; doi:10.1136/bmjopen-2025-100361)
Supplement: online supplemental file 1 [file bmjopen-15-9-s001.docx]

**Supplementary material – PLOT-ICH Case report form**

**Initial presentation and admission data**

| ORION unique patient identifier |  |
| --- | --- |
| Sex | Male / Female |
| Age (in years at time of admission) | yyy |
| Date and time of symptom onset | dd/mm/yyyy hh:mm  [with option for unknown] |
| Presenting symptoms [*select all that apply*] | - Collapse - Headache - Focal neurological deficit (weakness, facial asymmetry, sensory changes) - Speech disturbance - Seizure |
| Date / time of admission to hospital | dd/mm/yyyy hh:mm |
| Was the patient directly transferred from presentation to your hospital? | Yes / No |
| Hospital patient was transferred from | _____________ |
| Method of transport to your institution | - Helicopter (air ambulance) - Land ambulance (staffed by paramedics) - Land ambulance (not staffed by paramedics) - Police - Private vehicle - By foot - Other |
| American Society of Anaesthesiologists (ASA) physical status classification | I II III IV V |
| Glasgow Coma Score on admission (or last documented before sedation if sedated on admission) | \| Eye response \| Verbal response \| Motor response \| \| --- \| --- \| --- \| \| 1 \| 1 \| 1 \| \| 2 \| 2 \| 2 \| \| 3 \| 3 \| 3 \| \| 4 \| 4 \| 4 \| \|  \| 5 \| 5 \| \|  \| T (intubated) \| 6 \|   Total GCS _____ |
| Pupillary response on admission | \|  \| Right \| Left \| \| --- \| --- \| --- \| \| Reactive \|  \|  \| \| Fixed and dilated \|  \|  \| \| Small and unreactive \|  \|  \| \| Unknown \|  \|  \| |
| NIHSS on admission (<https://www.mdcalc.com/calc/715/nih-stroke-scale-score-nihss#when-to-use>) | _________  *[with option for unknown]* |
| WFNS scale on admission (if SAH) | 1. GCS 15 2. GCS 13-14 without a focal motor deficit 3. GCS 13-14 with a focal motor deficit 4. GCS 7-12 5. GCS 3-6 |
| Known comorbidities [*select all that apply*] | - none - hypertension - atrial fibrillation - diabetes - family history of intracranial aneurysms - obesity - HIV - previous stroke - bleeding disorder - sickle cell |
| Known medication history | - Antiplatelet use: Yes, No, Unknown - Anticoagulant use: Yes, No, Unknown   If Yes to Anticoagulant use, choose which anticoagulant:   - Warfarin - Direct oral anticoagulants (DOAC) - Heparins |
| Body mass index | ____ |
| Smoking status | Yes, No, Ex-smoker, Unknown |
| Illicit drug use (e.g. cocaine, amphetamines) | Yes, No, Unknown |
| Alcohol use | Yes, No, Unknown |
| Admission location | - general ward - stroke unit - neurology ward - neurosurgery ward - HDU - ICU |
| Admitting team | - Emergency medicine - General medicine - Stroke team - Neurology team - Neurosurgery team - Intensive care team |

**Imaging data**

| Date and time of first imaging available for review | dd/mm/yyyy hh:mm |
| --- | --- |
| First imaging performed at your institution? | Yes / No |
| First imaging modality | - Computed tomography - Magnetic resonance imaging - X-ray - Ultrasound |
| What is the primary intracranial haemorrhage type? | - Subarachnoid haemorrhage - Intraparenchymal haemorrhage - Intraventricular haemorrhage |
| *If ‘intraparenchymal haemorrhage’ selected* | |
| Lateralisation | Right / Left / Multifocal / Midline |
| Midline shift | - No - 1-5 mm - 6-10 mm - >10 mm |
| Intraventricular extension of haemorrhage | Yes / No |
| Subarachnoid extension of haemorrhage | Yes / No |
| Basal cisterns | - Open - Compressed / obliterated |
| Location | - Cortical / lobar - Basal ganglia / internal capsule / thalamus - Pons / brainstem - Cerebellum |
| Hydrocephalus present | Yes / No |
| Volume of intracerebral haemorrhage (ABC/2 formula | - Unknown - 0-15 mL - 15-30 mL - 30-60 mL - >60 mL |
| ICH score (<https://www.mdcalc.com/calc/402/intracerebral-hemorrhage-ich-score>) | ___ |
| sICH score (<https://www.mdcalc.com/calc/3802/secondary-intracerebral-hemorrhage-sich-score>) | ___ |
| *If ‘subarachnoid haemorrhage’ selected* | |
| Location of SAH | Diffuse / Focal |
| Intraventricular extension of haemorrhage | Yes / No |
| Intraparenchymal extension of haemorrhage | Yes / No |
| Hydrocephalus present | Yes / No |
| Basal cisterns | - Open - Compressed / obliterated |
| Modified Fisher scale | 1. Thin SAH (<1 mm), no IVH 2. Thin SAH, IVH present 3. Thick SAH, no IVH 4. Thick SAH, IVH present |
| *If ‘intraventricular haemorrhage’ selected:* | |
| Location of haemorrhage [*select all that apply*] | - Right lateral ventricle - Left lateral ventricle - Third ventricle - Fourth ventricle |
| Graeb score | _____ |
| Hydrocephalus present | Yes / No |
| Basal cisterns | - Open - Compressed / obliterated |
| *Advanced imaging:* | |
| Did the patient have vascular imaging? | - Yes / No   If Yes, which type:   - Cerebral angiogram (DSA) - CT angiogram - MR angiogram   If Yes, at your institution? Yes / No |
| Did the patient have other advanced imaging? | - Yes / No   If Yes: CT + contrast / MRI + contrast / Other  If Yes, at your institution? Yes / No |
| Did further imaging identify an underlying structural cause for the intracranial haemorrhage? [*select all that apply*] | Yes / No / NA  If Yes, specify:   - Aneurysm - Arteriovenous malformation - Dural arteriovenous fistula - Cavernoma - Tumour - Venous sinus thrombosis - Infection - Moyamoya / vasculopathy / vasculitis |

**Acute management data**

| Was the patient hypertensive on admission? | Yes / No  If Yes, what range was the systolic blood pressure in:   - 140-160 mmHg - 160-200 mmHg - >200 mmHg   If Yes, how was hypertension managed:   - Oral or topical antihypertensives only - Intravenous antihypertensives needed   If Yes, was it normalised within 6 hours? Yes / No |
| --- | --- |
| Did the patient have invasive blood pressure monitoring e.g. arterial line? | Yes / No |
| Did the patient receive nimodipine? (if SAH) | Yes / No / NA |
| Did the patient have the following checked on admission? [*select all that apply*] | - Full blood count - Clotting - Renal function - Blood type - Blood glucose - Temperature |
| If the patient was taking anticoagulant medications, were these reversed on admission? | Yes / No / NA |
| Was the patient intubated on admission? | - Yes, prior to hospital admission - Yes, after arrival to hospital - No |
| Specialties consulted during admission [*select all that apply*] | - Emergency medicine - General medicine - Stroke team - Neurology team - Neurosurgery team - Intensive care team - Interventional radiology team |
| Did the patient receive input from therapy services during the admission? [*select all that apply*] | - Physiotherapy - Occupational therapy - Speech and language therapy - Neuropsychology |
| Did the patient undergo surgical intervention (including placement of an EVD or LD)? | Yes / No |
| Did the patient require transfer to a different acute hospital facility for ongoing care? | Yes / No  If Yes, what was the primary reason for transfer?   - Surgical intervention (includes endovascular) - Admission to HDU / ICU - Admission to neurosurgery unit - Admission to acute stroke unit |

**Operative data (if the patient underwent operative intervention at your institution)**

| Grade of the most senior surgeon present in the operating theatre | - Fully qualified neurosurgeon - Neurosurgeon in training - Other fully qualified surgeon - Other surgeon in training - Interventional radiologist / medical professional fully qualified in endovascular surgery - Medically qualified but not in a surgical training programme - Non-medically qualified surgical provider |
| --- | --- |
| Grade of the most senior anaesthesia provider present in the operating theatre | - Fully qualified anaesthetist with medical qualification - Anaesthetist in training with medical qualification - Not medically qualified anaesthesia provider - Anaesthetic administered by surgeon - No anaesthesia provided |
| Type of anaesthesia | General / Local / None |
| Date and time of operation | Dd/mm/yyyy HH:MM |
| Duration of operation | HH:MM |
| Were pre-incision prophylactic antibiotics given? | Yes / No / Unknown |
| Class of surgical wound | - Clean - Clean-contaminated - Contaminated - Dirty-infected |
| Location of surgery | Right / Left / Bilateral / Midline |
| What was the main procedure undertaken? | - Insertion of external ventricular drain (EVD) - Insertion of lumbar drain (LD) - Insertion of ventriculoperitoneal shunt - Supratentorial craniotomy / craniectomy - Evacuation of intracerebral haematoma (open)   - Minimally invasive evacuation of intracerebral haematoma   - Clipping of anterior circulation aneurysm   - Clipping of posterior circulation aneurysm   - Ligation of arterio-venous malformation   - Ligation of dural arteriovenous fistula   - Decompressive craniectomy (no haematoma evacuated)   - Direct or indirect revascularisation surgery - Infratentorial craniotomy / craniectomy   - Evacuation of posterior fossa haemorrhage   - Ligation of posterior fossa AVM / dAVF / vascular abnormality   - Clipping of posterior circulation aneurysm   - Posterior fossa decompression (no haematoma evacuated) - Endovascular treatment   - Coiling / stenting / embolisation of anterior circulation aneurysm   - Coiling / stenting / embolistion of posterior circulation aneurysm   - Embolization of AVM / dAVF / other vascular abnormality |
| *If ‘Insertion of EVD’ or ‘Insertion of LD’ was selected:* | Where was the EVD insertion or LD insertion procedure performed?   - Operating theatre - Patient bedside |
| *If ‘Craniotomy’ was selected:* | What was done with the bone flap at the end of the procedure?   - Replaced and fixed - Replaced and left floating / hinged - Removed and placed in the abdomen - Removed and stored - Removed and discarded   Was duraplasty performed? Yes / No  If Yes:   - Apposition of dural edges and watertight closure - Rough approximation of dural edges but no watertight closure - Autologous graft with watertight closure - Autologous graft laid on top of dura with no watertight closure - Non-autologous graft with watertight closure - Non-autologous graft without watertight closure   Did the patient have a wound drain placed? Yes / No  Did the patient have an external ventricular drain inserted?   - Yes, for CSF diversion for hydrocephalus - Yes, for ICP monitoring alone - No   Was an intraparenchymal ICP monitor inserted? Yes / No  Was an operating microscope available and used in the operation?   - Available and used - Available but not used - Not available |
| Was image guidance available and used in the operation? | - Available and used - Available but not used - Not available |
| Intraoperative death | Yes / No |
| Further comments regarding the procedure |  |
| Did a second operation take place for primary treatment of the ICH? | Yes / No    If Yes, expand operative details again. |

**Outcome data**

| Death within the 30-day follow-up period? | Yes / No  If Yes:   - Date of death: dd/mm/yyyy |
| --- | --- |
| If ‘No’ to ‘death within the follow-up period’, was the patient still an inpatient at the end of the 30-day follow-up period? | Yes / No  If No:   - Date of discharge: dd/mm/yyyy - Discharge destination:   - Home   - Rehabilitation institution   - Other acute hospital facility   - Unknown |
| Was the patient admitted to intensive care during the 30-day follow up period? | Yes / No / Unknown  If Yes:   - Date of admission to ICU: dd/mm/yyyy - Was the patient discharged from ICU during the follow up period? Yes/ No   If Yes:   - Date of discharge from ICU: dd/mm/yyyy |
| Did the patient require intubation and ventilation during the admission? | Yes / No |
| Did the patient require a tracheostomy? | Yes / No |
| Did the patient require ventilatory support at the time of death / discharge / end of follow-up period? | Yes / No |
| Did any adverse events occur? [*select all that apply*] | - None - Pressure ulcer - Pneumonia - Pulmonary embolism - Deep vein thrombosis - Hyponatraemia - Re-bleed - Delayed cerebral ischaemia (DCI) - Hydrocephalus - Seizures   If ‘Delayed cerebral ischaemia’ selected, how was this diagnosed?   - Clinical diagnosis alone - Imaging (e.g. CT perfusion scan, angiogram)   If ‘Delayed cerebral ischaemia’ selected, how was it treated?   - No treatment - Intravenous hydration - Blood pressure augmentation - Angiographic treatment   If ‘Hydrocephalus’ selected, how was this treated?   - Lumbar puncture alone - Temporary CSF drainage e.g. EVD or lumbar drain - Permanent CSF diversion (e.g. VP shunt insertion) - Not treated   If ‘Seizures’ selected, how were they treated?   - No treatment - Antiepileptic medications |
| If the patient underwent surgery, did a surgical site infection occur? | - Yes, superficial (wound) infection - Yes, deep tissue (bone or brain) infection - No - NA   If Yes, how was it treated?   - Antibiotics only - Wound debridement - Removal of bone / intracranial washout |
| Did the patient return to the operating theatre during the admission (after the initial surgical management)? | - Yes, planned - Yes, unplanned - No   If Yes, specify operation: _____ |
| Modified Rankin scale (mRS) at 30 days or discharge (<https://www.mdcalc.com/calc/1890/modified-rankin-scale-neurologic-disability>) | - 0: no symptoms - 1: no significant disability - 2: slight disability - 3: moderate disability - 4: moderate severe disability - 5: severe disability - 6: dead |
| GCS at 30 days or discharge | \| Eye response \| Verbal response \| Motor response \| \| --- \| --- \| --- \| \| 1 \| 1 \| 1 \| \| 2 \| 2 \| 2 \| \| 3 \| 3 \| 3 \| \| 4 \| 4 \| 4 \| \|  \| 5 \| 5 \| \|  \| T \| 6 \|   GCS total __ |
