# Supplementary material for: Assessing the global variation in patient characteristics, management and short-term outcomes of spontaneous intracranial haemorrhage worldwide: a protocol for a global observational prospective multicentre study (the PLOT-ICH study)
Source: BMJ Open. 2025 Sep 2;15(9):e100361. doi: 10.1136/bmjopen-2025-100361 (PMC12406811; doi:10.1136/bmjopen-2025-100361)
Supplement: Supplementary file 2 [file bmjopen-2025-100361.reviewer_comments.pdf]

PEER REVIEW HISTORY

BMJ Open publishes all reviews undertaken for accepted manuscripts. Reviewers are asked to complete a checklist review form and are provided with free text boxes to elaborate on their assessment. These free text comments are reproduced below.

ARTICLE DETAILS

Title (Provisional)

Assessing the global variation in patient characteristics, management and short-term outcomes of spontaneous intracranial haemorrhage worldwide: a protocol for a global observational prospective multicentre study (the PLOT-ICH study)

Authors

Venturini, Sara; Clark, David; Smith, Brandon George; Hobbs, Laura; Bath, Michael F.; Mee, Harry; Still, Megan; Mediratta, Saniya; Soliman, Mohamed Amin; Kohler, Katharina; Whiffin, Charlotte Jane; Katambo, Elijah; Korhonen, Tommi K.; Tetri, Sami; Bankole, Nourou Dine Adeniran; Rutabasibwa, Nicephorus; Bhebe, Arnold; Munusamy, Thangaraj; Tirsit, Abenezer; Park, Kee B; Budohoski, Karol; Wahjoepramono, Petra; Tamborska, Arina; Saylor, Deanna; Khan, Tariq; Khattak, Almas Fasih; Figaji, Anthony; Kolias, Angelos G; Bashford, Tom; Joannides, Alexis; Martin, Michael; Trivedi, Rikin A; Hutchinson, Peter John; the PLOT-ICH Collaborative, on behalf of

---

VERSION 1 - REVIEW

**Reviewer**	**1**
**Name**	**Kirshner , Howard**
**Affiliation**	**Vanderbilt University Medical Center**
**Date**	**20-Mar-2025**
**COI**	**None**

The proposed study is a good effort to get comparative data on management of cerebral hemorrhage from countries worldwide. I have a few specific questions:

I am not sure why combining ICH and SAH is useful. Should they be separate surveys? The treatment of each type of hemorrhage is quite different.

A 30-day study period seems very short. Some rural hospitals may go a month without seeing a hemorrhage patient. Should these centers be excluded?

The outcome of cerebral hemorrhage is often not clear at 30 days. Patients often improve for 6 months or more. I think at least a qualitative data point at longer than one month would be extremely helpful.

Rehabilitation for hemorrhagic stroke is likely to differ from country to country and from urban to rural settings. Should that be included as a data point?

There are a number of typographical/grammatical errors in the proposal.

**Reviewer**	**2**
**Name**	**Guo, Jianwen**
**Affiliation**	**Guangdong Provincial hospital of Chinese medicine, Neurology**
**Date**	**09-Jun-2025**
**COI**	**None**

Evaluation and Deficiency Analysis

1. **Study Design and Methodology**

- **Strengths**:

- The study adopts a prospective, multicenter, international observational cohort design, covering high-, middle-, and low-income countries, ensuring strong data diversity.

- A 30-day short-term follow-up reduces loss-to-follow-up rates and is suitable for global collaboration.

- Feasibility was validated through a pilot study, demonstrating methodological rigor.

- **Deficiencies**:

- **Sample Size Calculation**: Based on conservative mortality estimates (30% in HICs vs. 40% in LMICs), 712 patients are required. *using an alpha value of 0.05 and power of 80%*- why use power of 80% not 90%?

- **Handling of Missing Data**: Only 70% field completeness is required, which may lead to missing key variables (e.g., imaging results) and compromise analysis quality.

- **Lack of Long-Term Outcomes**: Focusing solely on 30-day outcomes prevents assessment of functional recovery or long-term survival rates, limiting a comprehensive understanding of disease burden.

2. **Ethics and Data Management**

- **Strengths**:

- Approved by the University of Cambridge Ethics Committee, complying with international standards. Data is encrypted and stored via the ORION platform, meeting NHS security requirements.

- Explicit anonymization of patient data ensures privacy protection.

- **Deficiencies**:

- **Variability in Local Ethics Approvals**: No mention of how differences in national ethics requirements will be reconciled, potentially delaying participation from some centers.

- **Insufficient Data Sharing Details**: Unclear whether collaborative centers can access aggregated data or conduct secondary analyses.

3. **Study Implementation**

- **Strengths**:

- Recruitment leverages existing research networks (e.g., NIHR), enhancing feasibility.

- Patient and Public Involvement (PPI) influenced study design, improving result relevance.

- **Deficiencies**:

- **Inadequate Consideration of Resource Disparities**: High-income countries may more easily provide advanced imaging (e.g., CTA/MRA), while LMICs rely on CT, leading to diagnostic bias.

- **Lack of Surgical Technique Standardization**: No description of how surgical details (e.g., definition of "minimally invasive hematoma evacuation") are uniformly recorded, potentially introducing operational heterogeneity.

4. **Outcome Measures**

- **Strengths**:

- Primary outcome (30-day mortality) is clear, and secondary outcomes (GCS, mRS, hospital stay duration) cover key clinical indicators.

- **Deficiencies**:

- **Functional Assessment Limitations**: mRS and GCS evaluations at discharge may be influenced by acute-phase effects, and more sensitive scales (e.g., Barthel Index) are not included.

5. **Statistical Analysis**

- **Strengths**:

- Planned multivariate logistic regression controls for confounders, and stratification by HDI enhances comparability.

- **Deficiencies**:

- **No Mention of Multiple Testing Correction**: Multi-group comparisons (e.g., HICs vs. LMICs) may increase false-positive risks, necessitating adjustments like Bonferroni correction.

- **Unspecified Handling of Missing Data**: E.g., whether multiple imputation or sensitivity analysis will be used.

Overall, the study design is sound and globally significant, but further refinement in implementation details and data analysis is needed to ensure reliability and generalizability of results.

VERSION 1 - AUTHOR RESPONSE

Reviewer 1 comments

The proposed study is a good effort to get comparative data on management of cerebral hemorrhage from countries worldwide. I have a few specific questions:

I am not sure why combining ICH and SAH is useful. Should they be separate surveys? The treatment of each type of hemorrhage is quite different.
We are including both to address global variation, this is also done in the GBD and similar studies. However, subgroup analyses based on the type of haemorrhage will also be performed to understand such differences. This has been specified in page 8 (statistical analysis section).

A 30-day study period seems very short. Some rural hospitals may go a month without seeing a hemorrhage patient. Should these centers be excluded?
Any centre including at least one patient in the 30-day period will be included in the study. The same data collection duration has been chosen for each participating site to ensure equal contribution to the study; the differences in case volumes between hospital types will be explored and described in the results.

The outcome of cerebral hemorrhage is often not clear at 30 days. Patients often improve for 6 months or more. I think at least a qualitative data point at longer than one month would be extremely helpful.
We are aware that 30-day outcomes are short term outcomes in brain haemorrhage, The reason for this choice is the pragmatic design of this study. We also based our decision on other studies conducted by our research team and other teams, with similar methodologies (namely the GNOS study – global neurotrauma outcomes study, which used a 2-week outcome, the GOAL-Trauma study, and the GloabSurg and CovidSurg collaborative studies). The aim of this study is to describe and assess global variation, and not to provide detailed information on long-term patient outcomes. Although a longer outcome time point would offer useful information, this would increase the workload for all collaborating sites, and possibly limit the ability to complete the study fully. Specifically, we are only collecting data for the duration of time patients are in hospital during the index admission, and a 6 month follow up point would require significant more resources to contact patients following hospital discharge. This limitation has been clearly acknowledged in the Limitations section.

Rehabilitation for hemorrhagic stroke is likely to differ from country to country and from urban to rural settings. Should that be included as a data point?
Rehabilitation outcomes (long term) is not being investigated due to the pragmatic nature of

the study, as described in the answer above. However, we are asking about access to rehabilitation services (physiotherapy, occupational therapy, etc in the short term acute phase) in the site questionnaire component of the study, to provide institution-level information of capacity for longer-term care for patients with brain haemorrhage.

There are a number of typographical/grammatical errors in the proposal.
Thank you, these have been corrected in the manuscript. British English spelling has been used throughout.

Reviewer 2 comments

Evaluation and Deficiency Analysis
1. **Study Design and Methodology**
Strengths:
The study adopts a prospective, multicenter, international observational cohort design, covering high-, middle-, and low-income countries, ensuring strong data diversity.
A 30-day short-term follow-up reduces loss-to-follow-up rates and is suitable for global collaboration.
Feasibility was validated through a pilot study, demonstrating methodological rigor.
Thank you for highlighting these. No changes have been made to the manuscript.

Deficiencies:
Sample Size Calculation: Based on conservative mortality estimates (30% in HICs vs. 40% in LMICs), 712 patients are required. *using an alpha value of 0.05 and power of 80%*- why use power of 80% not 90%?
Thank you. We considered this point within the entire study steering committee, and chose 80%, which is standard for large observational studies. This is in line with the pragmatic nature of the study.

Handling of Missing Data: Only 70% field completeness is required, which may lead to missing key variables (e.g., imaging results) and compromise analysis quality.
We acknowledge that there will be some missing data. However, this study was designed to be pragmatic in nature and adopt a real-world design. The study team discussed balancing a more stringent cutoff with a more complete dataset, which would likely result in having to exclude a larger number of patients entered, with accepting a degree of missing data but with a final dataset that is more representative of real-world patient population. Based on previous similar studies (GNOS), we decided to accept this cutoff to achieve this balance. This has been added to the Limitations sections of manuscripts.

Lack of Long-Term Outcomes: Focusing solely on 30-day outcomes prevents assessment of functional recovery or long-term survival rates, limiting a comprehensive understanding of disease burden.
Thank you for the comment regarding long-term follow-up. Please also see the answer provided above to Reviewer 1’s comment regarding the same point. This study does not aim to describe long-term outcomes of haemorrhagic stroke, but only short-term outcomes. The methodology for this is based on previous studies conducted with similar methods, including

the GNOS spine on TBI, the GlobalSurg studies, and the currently running GOAL-Trauma study. Achieving long-term follow-up data with good completion rates and low dropout rates would be challenging in such a diverse global population. Therefore, a pragmatic approach was taken.

2. **Ethics and Data Management**
Strengths:
Approved by the University of Cambridge Ethics Committee, complying with international standards. Data is encrypted and stored via the ORION platform, meeting NHS security requirements.
Explicit anonymization of patient data ensures privacy protection.
Thank you for highlighting these. No changes have been made to the manuscript.

Deficiencies:
Variability in Local Ethics Approvals: No mention of how differences in national ethics requirements will be reconciled, potentially delaying participation from some centers.
Thank you. This has been considered at length in the set-up of the study. We obtained overall approval for the study itself at the University of Cambridge. The study documents and protocol outline the process each site will undertake to sign up. We are aware that ethical approval processes vary across countries, and this has been accounted for. We have prepared sample documents that sites can utilise to obtain local clearance. Each site will have to undergo their local approval processes and provide confirmation of approval before any patient data are entered. We have accounted for time taken to set this up in the study timeline. The study coordinator will keep track of each site progress and assist as needed. This has been clarified in the Ethics and dissemination section.

Insufficient Data Sharing Details: Unclear whether collaborative centers can access aggregated data or conduct secondary analyses.
Data sharing agreements are signed by each participating site with the coordinating institution. Each participating site will automatically have access to their own site data for local reporting and analysis. Aggregated data will be made available to sites asking for any reasonable request for post-hoc analyses, after the primary analysis is completed.

3. **Study Implementation**
Strengths:
Recruitment leverages existing research networks (e.g., NIHR), enhancing feasibility.
Patient and Public Involvement (PPI) influenced study design, improving result relevance.
Thank you for highlighting these. No changes have been made to the manuscript.

Deficiencies:
Inadequate Consideration of Resource Disparities: High-income countries may more easily provide advanced imaging (e.g., CTA/MRA), while LMICs rely on CT, leading to diagnostic bias.
Thank you. We are collecting detailed information about the resources available at each participating site, to correlate these with the patients recruited into the study and specifically describe such global variation. The limitations section acknowledges that there will be patients who are not included in the study due to diagnostic deficiencies and limitation. In the study manuscript, this will be clearly described in the results and discussion session.

**Lack of Surgical Technique Standardization**: No description of how surgical details (e.g., definition of "minimally invasive hematoma evacuation") are uniformly recorded, potentially introducing operational heterogeneity.
A detailed glossary of terms to assist completion of data field is provided to all participating sites and teams, to ensure uniformity in the terms and definitions used. This is available for review at the study website www.plotich.org/study-documents/ . Full details have not been included in the manuscript due to brevity, but a link to the study website is provided in the abstact and main text.

4. **Outcome Measures**
**Strengths**:
Primary outcome (30-day mortality) is clear, and secondary outcomes (GCS, mRS, hospital stay duration) cover key clinical indicators.
Thank you for highlighting these. No changes have been made to the manuscript.

**Deficiencies**:
**Functional Assessment Limitations**: mRS and GCS evaluations at discharge may be influenced by acute-phase effects, and more sensitive scales (e.g., Barthel Index) are not included.
As addressed in previous comments, the choice of outcome measures was discussed with the study steering committee as well as patient groups and is in line with the pragmatic nature of the study. Utilising more complex metrics can be challenging on such a global scale in a large number of sites.

5. **Statistical Analysis**
**Strengths**:
Planned multivariate logistic regression controls for confounders, and stratification by HDI enhances comparability.
Thank you for highlighting these. No changes have been made to the manuscript.

**Deficiencies**:
**No Mention of Multiple Testing Correction**: Multi-group comparisons (e.g., HICs vs. LMICs) may increase false-positive risks, necessitating adjustments like Bonferroni correction.
Thank you. Adjustments will be used in the analysis according to the full statistical analysis plan. These have not been included in the manuscript due to word count, but will be included in full in the main study manuscript.

**Unspecified Handling of Missing Data**: E.g., whether multiple imputation or sensitivity analysis will be used.
As discussed in the manuscript, data validation will be used to ensure data robustness and completion. However, a degree of missing data is expected in such a large international study. The pragmatic nature of the dataset, limiting the number of data points to essential ones, has been chosen to limit the impact of missing data on the key outcomes. Missing data will clearly be indicated and acknowledged in the main results of the study.

Overall, the study design is sound and globally significant, but further refinement in

implementation details and data analysis is needed to ensure reliability and generalizability of results.
Thank you for highlighting these. No changes have been made to the manuscript.

VERSION 2 - REVIEW

**Reviewer**	**2**
**Name**	**Guo, Jianwen**
**Affiliation**	**Guangdong Provincial hospital of Chinese medicine, Neurology**
**Date**	**10-Aug-2025**
**COI**	

1、The study protocol emphasizes a 30-day follow-up for pragmatic reasons, balancing feasibility with global participation. Extending follow-up would require additional resources and increase attrition risks.

2、Sample size calculations (n=712) are based on conservative mortality estimates (30% HICs vs. 40% LMICs) with 80% power, but subgroup analyses by income level are planned.

3、Site questionnaires capture hospital resources and care processes, allowing stratification by facility capability independent of national income.

4、Missing data handling (70% completeness threshold) and multivariable regression are specified to address potential imbalances.

5、Key Considerations for Implementation:

（1）Follow-up Extension: Would require protocol amendments and additional ethical approvals, potentially limiting LMIC participation.

（2）Statistical Balancing: Propensity score matching or inverse probability weighting could be employed post-hoc if imbalances arise.

（3）Baseline Severity Adjustment: Collected variables (GCS, ICH score, imaging findings) will enable case-mix adjustment in analyses.

（4）Provider-Level Variability: The site questionnaire explicitly documents center-specific capabilities (e.g., advanced imaging availability, surgical expertise) to contextualize outcomes.

VERSION 2 - AUTHOR RESPONSE

Reviewer 2 comments

The study protocol emphasizes a 30-day follow-up for pragmatic reasons, balancing feasibility with global participation. Extending follow-up would require additional resources and increase attrition risks.
Thank you. This is a comment confirming our answer to the previous queries raised, rather than a new question, therefore no changes have been made to the manuscript.

Sample size calculations (n=712) are based on conservative mortality estimates (30% HICs vs. 40% LMICs) with 80% power, but subgroup analyses by income level are planned.
Thank you. This is a comment confirming our answer to the previous queries raised, rather than a new question, therefore no changes have been made to the manuscript.

Site questionnaires capture hospital resources and care processes, allowing stratification by facility capability independent of national income.
Thank you. This is a comment confirming our answer to the previous queries raised, rather than a new question, therefore no changes have been made to the manuscript.

Missing data handling (70% completeness threshold) and multivariable regression are specified to address potential imbalances.
Thank you. This is a comment confirming our answer to the previous queries raised, rather than a new question, therefore no changes have been made to the manuscript.

Key Considerations for Implementation:

1. *Follow-up Extension: Would require protocol amendments and additional ethical approvals, potentially limiting LMIC participation.*
2. *Statistical Balancing: Propensity score matching or inverse probability weighting could be employed post-hoc if imbalances arise.*
3. *Baseline Severity Adjustment: Collected variables (GCS, ICH score, imaging findings) will enable case-mix adjustment in analyses.*
4. *Provider-Level Variability: The site questionnaire explicitly documents center-specific capabilities (e.g., advanced imaging availability, surgical expertise) to contextualize outcomes.*

Thank you. These points raised are comments rather than direct questions requiring change to the manuscript. The limitations highlighted are clearly stated in the ‘Strengths and limitations of this study’ section, as well as the Limitations section in the discussion.
